# Loop mediated isothermal amplification (LAMP) as a rapid and portable diagnostic tool for the detection of pea root rot pathogens

**DOI:** 10.1038/s41598-025-18738-9

**Published:** 2025-10-07

**Authors:** Alba Pacheco-Moreno, Samuel Bruty, Henry Tidd, Eyerusalem Morka, Sanu Arora

**Affiliations:** 1 Department of Biochemistry and Metabolism, John Innes Centre, Norwich Research Park, Norwich, UK; 2 Department of Crop Genetics, John Innes Centre, Norwich Research Park, Norwich, UK; 3 NIAB, Cambridge, CB3 0LE UK

**Keywords:** Biological techniques, Molecular biology, Plant sciences

## Abstract

Pea root rot, caused by soilborne pathogens such as *Aphanomyces euteiches*, various *Fusarium* species and *Pythium ultimum*, represents a significant challenge to global pea production. Current diagnostic methods are costly, time-consuming and require specialised laboratory setups, limiting their application for rapid decision-making in agricultural settings. To address this, we developed a Loop-Mediated Isothermal Amplification (LAMP) assay targeting the Internal Transcribed Spacer 1 (ITS1) region of these pathogens for rapid and in-field detection. The assay demonstrated high specificity and sensitivity, with detection limits as low as 0.02 ng of gDNA and an initial inoculum of 10 spores per sample in both soil and infected root tissue. Results were generated within 60 min, providing a practical alternative to conventional diagnostic methods. Validation with qPCR showed LAMP to be comparable or superior in accuracy, and field deployment with a portable LAMP device confirmed its effectiveness for on-farm diagnostics. This LAMP assay offers a robust, cost-effective and user-friendly tool for the early detection of pea root rot pathogens, enhancing disease management and potentially reducing crop losses.

**Supplementary Information:**

The online version contains supplementary material available at 10.1038/s41598-025-18738-9.

## Introduction

Pea is a valuable crop, recognised for its role in sustainable agriculture and nutritional benefits for both human consumption and animal feed. It improves soil health by fixing atmospheric nitrogen through its interaction with *Rhizobium* bacteria, thereby reducing the fertilizer requirements. Furthermore, pea serves as an excellent source of plant-based protein, offering a more sustainable alternative to animal protein due to its lower carbon footprint^1^. It is also increasingly preferred as a substitute for soy protein. With growing consumer awareness, the pea protein market is projected to achieve a compound annual growth rate (CAGR) of about 13% between 2022 and 2030^2^. However, growers face significant challenges with pea cultivation, as yield volatility makes investment in the crop risky. A major constraint on global pea production is the prevalence of soil-borne root pathogens that can co-occur and infect different parts of the root system, leading to its decay and subsequently impacting plant health and causing severe yield losses.

Pea root rot is caused by a complex of fungal and oomycete pathogens that often act synergistically. The causal organisms include *Aphanomyces euteiches*, *Pythium* spp. (including *Pythium ultimum*), and *Fusarium* spp., particularly *F*. *solani*, *F*. *avenaceum*, *F*. *oxysporum* and *F*. *redolens*^3,4^. The dominant pathogens within this complex can vary by geographical region and are influenced by environmental factors such as moisture, temperature, pH, soil compaction, host preference and agricultural practices. For instance, a study conducted in major pea growing regions of Canada reported that while *Fusarium* spp. are widely distributed, *F. solani* and *F. avenaceum* are among the most virulent. Meanwhile, *Aphanomyces*, which was detected in Canada in 2012, has become quite widespread in the pea fields and causes severe root rot under conditions of free soil moisture^5^. Both *Fusarium* and *Aphanomyces* often co-occur within the root rot complex, and may act synergistically to cause more severe symptoms^6^. Although *Pythium* spp. are generally considered less aggressive but are also an important contributors to the root rot complex, particularly under cool, wet conditions where they can cause significant early-season seedling damping-off and root decay^7^. This can result in delayed growth, reduced nodulation and potentially leading to yield loss due to poor establishment. While *Pythium* spp. are primarily considered seedling pathogens, they can also affect later stages of root development.

These pathogens form resilient spores with thick cell walls which can survive in the soil for decades even in the absence of a host. When a susceptible host is planted, these spores germinate and infect the host roots. The disease is observed as yellow patches in the field with affected plants exhibiting stunting, yellowing, or wilting symptoms in the aerial parts while the root system shows significant constriction and the characteristic honey-colour, with black lesions extending up to the base of the stem^8^.

There are currently limited seed treatments and no complete genetic resistance available to combat the root rot complex. The primary management strategy involves testing soil infestation levels prior to planting. For highly infested fields, it is recommended to either avoid planting peas altogether or extend crop rotation for up to six to eight years. In fields with low to moderate infestation levels, growers may choose to plant either winter peas or avoid using susceptible cultivars for spring sowing. The soil’s inoculum potential is typically assessed using a baiting method^9,10^ in which peas are grown in pots with soil collected from the infected field and maintained under favourable conditions for disease development. After 5–6 weeks, the plants are evaluated for any root discolouration on a scale of 1–5. Several modifications of this method and culture-based techniques have been developed^11^, they often require significant time, and are both labour and space intensive.

Recently, advanced molecular methods have been introduced to improve detection sensitivity and specificity by targeting conserved regions in pathogen genomes. These include quantitative polymerase chain reaction (qPCR) assays targeting multicopy internal transcribed spacer 1 (ITS1) region of ribosomal DNA operons^12^ or translation elongation factor 1-α gene (TEF1)^13^. These methods have been adapted into multiplex qPCR for detection of both *Fusarium* and *Aphanomyces*^6^, and droplet digital PCR (ddPCR) has been employed to quantify low inoculum levels in soil. Using this method, a relationship was established between oospore density and disease severity in plants^14^. Whilst these molecular methods are good at predicting soil inoculum potential, they are expensive, time-consuming and require sending soil samples to a specialised testing facility. Our aim is to develop a rapid, on-farm and cost-effective (<£10/sample) testing system that is easily accessible to growers.

To address the limitations of current diagnostic methods, alternative molecular techniques like Loop-Mediated Isothermal Amplification (LAMP) have gained significant attention in recent years and are now available in portable formats^15^. LAMP is an isothermal DNA amplification technique that offers high specificity, sensitivity, and efficiency. It presents several advantages over conventional PCR, including simplicity, rapidity, and robustness. Unlike PCR, LAMP does not require sophisticated and expensive thermal cyclers as it can be performed using basic equipment such as a water bath or a heating block. Additionally, measuring amplification within a LAMP reaction is straightforward, with options ranging from colorimetric indicators to fluorescent dyes, enabling real-time detection. LAMP’s high specificity arises from its use of multiple primers annealing to distinct regions within the target DNA, significantly reducing the likelihood of non-specific amplification. Furthermore, the strand displacement activity of DNA polymerases used in the LAMP reaction enhances its efficiency and specificity. Its isothermal nature allows for rapid amplification, typically within 30–60 min, enabling on-site and real-time detection, which is particularly advantageous for disease management decisions. LAMP has demonstrated superior performance in the detection of various plant pathogens^16,17^. This study focuses on the design and evaluation of LAMP primers for the specific detection of pea root rot pathogens, *A. euteiches*, *P. ultimum*, *F*. *solani*, and *F*. *oxysporum*. By targeting the highly variable ITS1 region of pathogen genomes, we have developed a robust diagnostic tool that leverages the advantages of LAMP technology. The successful implementation of this LAMP assay as a portable device would provide farmers and agronomists with a valuable tool for early and accurate diagnosis of root rot pathogens, enabling timely disease management interventions and ultimately reducing the associated economic losses.

## Materials and methods

### Plant material

*Pisum sativum* cv. Ambassador, provided by van Waveren Saaten Seeds (Germany), was used throughout this study for all *in-planta* experiments. The seeds were surface sterilised using 70% ethanol for 1 min, followed by a 2-minute immersion in a 5% sodium hypochlorite solution. Subsequently, the seeds were rinsed up to six times with sterile deionised water. The sterilised seeds were then placed on 1.5% water agar plates and incubated in the dark at 25 °C for three days to allow germination.

### Microbial isolates collection

All fungal and oomycete isolates used in this study are listed in Table 1. A subset of these isolates were obtained as pure cultures from various sources, while others were isolated from the roots of pea plants grown in soil collected from an agricultural pea field in Reepham, Norfolk, UK (Table 1, Supplementary Fig. 1). To test the specificity and sensitivity of the LAMP test, additional isolates from other species were included. These species were *Fusarium redolens*, *Fusarium acuminatum*, *Phoma medicaginis* and *Rhizoctonia solani*, and other common soil organisms such as *Mucor hiemalis* and *Mortierella* spp.

**Table 1:** Fungal and oomycete isolates used in this study.

Isolate name	Species	Origin
*AeD*	*Aphanomyces euteiches* Drechsler	BCCM/MUCL Agro-food & Environmental Fungal Collection
*AeRB84*	*Aphanomyces euteiches* RB84	INRAE, IGEPP^18^
*Pul*	*Pythium ultimum var.* ultimum	NIAB
*FsolNIAB*	*Fusarium solani _*NIAB	NIAB
*FoxyNIAB*	*Fusarium oxysporum*_NIAB	NIAB
*FoxyR1*	*Fusarium oxysporum* Race 1	SASA
SA7	*Phoma medicaginis*	NIAB
SA9	*Rhizoctonia solani*	NIAB
SA13	*Mucor hiemalis* _MBV01222	This work^a^
SA17	*Mortierella* sp._MBV01228	This work^a^
SA27	*Fusarium redolens*_MGA01222	This work^a^
SA39	*Mucor hiemalis*_MBV01225	This work^a^
SA49	*Fusarium acuminatum*_MGA04221	This work^a^
SA59	*Fusarium solani*_MGA04227	This work^a^
SA73	*Fusarium solani*_MGA042215	This work^a^
SA77	*Fusarium oxysporum*_MGA042218	This work^a^
SA87	Fusarium solani_MBV04223	This work^a^
SA101	*Fusarium solani*_MGA052211	This work^a^
SA105	*Fusarium solani*_MGA052213	This work^a^
SA119	*Fusarium redolens*_MGA052220	This work^a^

^a^All isolates used in this study were obtained from visibly diseased pea roots grown in soil from an agricultural pea field in Reepham, Norfolk, UK.

### Isolation of genomic DNA

For microbial gDNA extraction, liquid cultures were grown in 10 ml of Potato Dextrose Broth (PDB) (Formedium, UK) with one actively growing mycelial plug. After 3–5 days, the cultures were centrifuged, the supernatant was removed, and approximately 250 mg of mycelial pellet was crushed in liquid nitrogen using a pestle and mortar or with glass beads in a TissueLyser II (QIAGEN, Germany).

When roots were used as the starting material, the entire root system was crushed in liquid nitrogen and 250 mg of tissue were taken for DNA extraction. The disrupted tissue was then processed using the DNeasy PowerSoil Pro kit (QIAGEN, Germany), following the manufacturer’s instructions. The quality (A_260_/A_280_) and quantity of the isolated DNA samples were evaluated using Nanodrop 2000 spectrophotometer (Thermo Fisher Scientific, USA) and Qubit fluorometer (Invitrogen, USA).

### Design of LAMP primers

The internal transcribed spacer 1 (ITS1) region was selected for designing LAMP primers to detect members of the root rot complex, namely *A. euteiches*,* P. ultimum*,* F. solani* and *F. oxysporum.* This region has been widely used for successful fungal identification, offering high resolution for distinguishing inter- and intraspecific variation^19,20^. To identify unique putative regions for each pathogen, 11 to 25 ITS sequences per target were downloaded from the NCBI database, including both f. sp. *pisi* and general species^21^. Given that a reasonably high sequence identity is expected, sequences were chosen based on their length (more than 600 bp) and availability at the time of analysis. These sequences were individually aligned using Geneious Prime 2025.0.3, generating a consensus sequence of 300–400 bp for each organism (Supplementary Fig. 2, 3 and Supplementary File 1). The consensus sequences were then used as input for the NEB LAMP primer design tool (https://lamp.neb.com/#!/). The uniqueness of primer sequences was checked using *blastn-short* in the NCBI database to confirm minimal off-target binding. The top 10 blastn hits can be found in Supplementary Table 1.

**Table 2:** LAMP primer sequences designed in this study using ITS genes of *F. solani*, *F. oxysporum*, *A*. *euteiches* and *P*. *ultimum*.

Pathogen	Primers	Sequence (5’-3’)
*F. solani*	F3_Fsol	GCCGTAAAACACCCAACTTC
	B3_Fsol	CGTTCCAGGGAACTCGGA
	FIP_Fsol	TGGGGCAATCCCTGTTGGTTTCTCAGGTAGGAATACCCGCTG
	BIP_Fsol	TTGAAATCTGGCTCTCGGGCCAGGCACCTCACCAAAAGC
*F. oxysporum*	F3_Foxy	TTTCAACAACGGATCTCTTG
	B3_Foxy	AATTAACGCGAGTCCCAA
	FIP_Foxy	TGATTCACTGAATTCTGCAATTCACCTGGCATCGATGAAGAACG
	BIP_Foxy	CCAGTATTCTGGCGGGCATGCACCAAGCTGTGCTTGAG
*A. euteiches*	F3_Apha	AAAACCATCCACGTGAATG
	B3_Apha	CAGTTCGCTGTGGTCTTC
	FIP_Apha	ATCGGTTCCTTGCGAAACCTTATTCTTTATGAGGCTTGTGC
	BIP_Apha	AACTAGCATCAGAAATGAAGCTTGTTGTGCGAGCCTAGACATC
*P. ultimum*	F3_Pul	ATTTATACTGTGGGGACGAA
	B3_Pul	AGAAAAAGAAAGGCAAGTTT
	FIP_Pul	CTTCATCGATGTGCGAGCCTAGGTCCTTGCTTTTACTAGATAACAAC
	BIP_Pul	ACGTAATGCGAATTGCAGAATTCAAGACATACTTCCAGGCATAA

### LAMP reaction setup and product visualisation

For the colorimetric LAMP reaction, a 10X primer mix containing all four primers was prepared. The FIP and BIP primers were prepared at a concentration of 16 µM, while F3 and B3 were prepared at 2 µM. The gDNA extracted from all samples was normalised to the same concentration, 20 ng/µl for roots and 5 ng/µl for soil. Additionally, 20 ng of pure target gDNA was included as a positive control. Each reaction contained 10 µl of WarmStart Colorimetric LAMP 2X Master Mix (NEB, USA), 2 µl of 10X primer mix and 1–5 µl of target DNA, with the final reaction volume adjusted to 20 µl using PCR grade water. For optimisation, different temperatures (65 and 66 °C) and reaction durations (35–60 min) were tested using a thermocycler. Results were visualised by observing colour changes with the naked eye, and high-resolution images were captured using a white lighting panel.

Additionally, fluorometric LAMP assays (RealAmp) were performed using the WarmStart Fluorescent LAMP/RealAmp Kit (NEB, USA) following the manufacturer’s standard protocol. Each reaction mixture contained 10 µl multi-purpose LAMP/RT-LAMP 2X Master Mix (NEB, USA), 2 µl of 10X primer mix, 0.4 µl of LAMP Fluorescent Dye 50X (NEB, USA), 1–5 µl of target DNA, with the final reaction volume adjusted to 20 µl using PCR grade water. The reaction was incubated at 66 °C for 60 min (as optimised for colorimetric assay). Fluorescent signals were recorded with the SYBR/FAM channel of a CFX96 Real-Time PCR Detection System (Bio-Rad, USA) and relative fluorescence units (RFU) were plotted against time.

### Evaluating the specificity and sensitivity of LAMP

The specificity of the LAMP assay was evaluated for all the primers sets across a set of 20 fungal and oomycete isolates (Table 1). The primer sets were tested by setting up a colorimetric LAMP reaction with 10 ng of microbial gDNA at 66 °C for 35–41 min. A positive LAMP reaction was indicated by a colour change from pink to bright yellow, whereas negative reactions remained pink.

To determine the minimum detectable concentration of the pathogens, a serial dilution of their gDNA ranging from 0.02 ng up to 40 ng was utilised as input for both the colorimetric and RealAmp LAMP assay at 66 °C for 35–41 min and 60 min, respectively.

### *In planta* severity assays and inoculum preparation

For *in planta* severity assays, different substrate mixes were used depending on the experimental setup. In the vermiculite-based assay, 25 ml glass tubes were filled with pre-wetted, medium-grade vermiculite, followed by 10 ml of modified Fahraeus medium (modFP) containing 1 mM CaCl_2_, 0.5 mM MgSO_4_, 0.7 mM KH_2_PO_4_, 0.8 mM Na_2_HPO_4_, 50 µM Fe EDTA and 0.1 mg/L of each of the following microelements: MnSO_4_, CuSO_4_, ZnSO_4_, H_3_BO_3_ and Na_2_MoO_4_, with the pH adjusted to 6. The entire system was autoclaved and stored at room temperature until use. For the autoclaved soil-based assay, a 1:1 mixture of the John Innes Centre’s cereal mix and sand was prepared and used to fill 50 ml tubes to a volume of 45 ml, after which 10 ml of distilled water was added. The tubes were then autoclaved and stored at room temperature until use. In the pot assay, 9 cm pots were filled with the same cereal mix (JIC) and sand mixture used in the autoclaved soil-based assay and were used immediately.

Three-day-old pea seedlings were transplanted into the 25 or 50 ml tubes and 5 ml of deinoised water was added after transplanting. In the pot assay, two seedlings were planted per pot. Plants were kept in a controlled growth chamber set at 25 °C during the day and 23 °C at night, with a 14-hour light/10-hour dark cycle. Watering was done twice weekly, with 5 ml added to the tubes and 30 ml added to the pots. Plants were inoculated at seven days post-planting with different combinations of *AeRB84* and *FsolNIAB.* To prepare *AeRB84* zoospores, 6–10 plugs of actively growing mycelium were transferred into 25 ml of PG medium (20 g/L peptone, 5 g/L glucose) and statically incubated in the dark at 25 °C for about 10 days. The mycelial mats were rinsed at 2-hour intervals with Volvic water and incubated for at least 16 h at 25 °C with shaking at 60 rpm. Zoospore concentrations were assessed the following day under a microscope using a hemocytometer and diluted to a final concentration of 10^5^ zoospores/ml. To prepare *FsolNIAB* conidia, 200 ml of PDB medium was inoculated with up to three plugs of actively growing mycelium and incubated in the dark at 25 °C for 5–7 days. Cultures were then filtered through two layers of sterile Miracloth, spores were counted under a microscope using a hemocytometer and the inoculum was diluted to a final concentration of 10^5^ conidia/ml using PBS (Formedium, UK) before setting up dilution series. For co-inoculation assays, *AeRB84* zoospores and *FsolNIAB* spores were mixed in a 1:1 ratio and 1 ml of the suspension was applied at the base of each plant. Plants were evaluated at 2 or 4 wpi and gDNA was extracted from both root and soil samples as described earlier.

### qPCR for detection of *F. solani* and *A*. *euteiches*

Previously published primers were used to detect the presence of *F.solani*^13^ and *A. euteiches*^12^ in pea plants inoculated during the disease severity assay. gDNA extracted from all samples was normalised to the same concentration: 20 ng/µl for roots and 5 ng/µl for soil. Additionally, 20 ng of pure gDNA from the target organism was included as a positive control. qPCR reactions were prepared using 2x qPCRBIO SyGreen Mix Lo-ROX (PCR Biosystems, UK) following the manufacturer’s instructions. The reactions were run with an annealing temperature of 60 °C with 40 amplification cycles, and data were acquired on the FAM channel using a CFX96 Real-Time PCR Detection System (Bio-Rad, USA).

### Soil sampling and root rot baiting

Field soil was collected from a site that exhibited severe pea root rot symptoms during the previous growing season near Peterborough (coordinates: 52.56765, 0.04128). Soil was sampled across the field in a W pattern, starting in the south-eastern corner, with samples taken approximately every 50 m. Using these soil samples, root rot soil baiting was carried out as described by Processors and Growers Research Organisation (PGRO)^22^. Briefly, sterilised pea seeds were germinated in the dark for five days before being transferred to petri dishes containing two layers of sterile filter paper. Fifteen grams of air-dried field soil was placed on one edge of each dish and 9 ml of sterile water was added. Seedlings were positioned such that their roots made contact with the soil while the seeds and shoots remained uncontaminated. The plates were sealed with micropore tape and incubated in the dark at 25 °C for 11–14 days. After incubation, seedlings were removed from the soil, washed with water and photographed. Root and/or soil samples were flash-frozen in liquid nitrogen for subsequent DNA extraction.

Microscope slides were prepared by cutting 0.5–1 cm pieces of root, ensuring a mix of primary and lateral roots. These sections were placed on a slide and crushed using another slide. A small volume of either water or trypan blue solution was added, a coverslip was placed on the top and slides were stored at 4 °C as they were not fixed. Microscopy was performed using a Zeiss Axio Imager in brightfield mode at 20x and 40x with air lenses. Images were captured using an AxioCam 506 colour camera with a colibri 7 light source. Post-capture processing was conducted using Fiji (ImageJ), including gamma correction (0.45), white balancing, and scale bar calibration.

### PEBBLE-based LAMP assay

A magnetic bead-based DNA extraction protocol was optimised for both soil and root tissue, following a modified version of the protocol described by Radhakrishnan et al.^23^. For this protocol, either 3 cm of root tissue or 250 mg of soil was used as the input material, along with 800 µL of lysis buffer. For roots, the tissue was disrupted with a mini pestle, followed by the addition of glass beads and vortexing for 5 min. For soil samples, glass beads were added directly to the soil before vortexing for 5 min. The resulting lysate was incubated in lysis buffer for 10 min and centrifuged at 13,000 g for 3–5 min. Subsequently, 500 µL of the supernatant was transferred into a fresh tube, and 50 µL of SeraSil-Mag™ 400 beads (Cytiva, USA) and 600 µL of binding buffer were added. The remaining steps of the protocol followed the original procedure without modification. gDNA was quantified using a Qubit fluorometer, with concentrations ranging from 1 to 6 ng/µL. The extracted gDNA was used directly for the LAMP assay.

The LAMP assay was conducted using the PEBBLE-R qcLAMP platform (BIOPIX DNA TECHNOLOGY P.C., Greece), a device capable of performing real-time colorimetric LAMP. WarmStart Colorimetric LAMP 2X Master Mix (NEB, USA) was used following the manufacturer’s standard protocol, with 5 µL of gDNA as input. Due to the design of the heating device in the PEBBLE platform, the reaction was incubated at an elevated temperature of 78–80 °C for 60 min to achieve 66 °C in the sample.

## Results

### Primer design and reaction optimisation

**Fig. 1:**
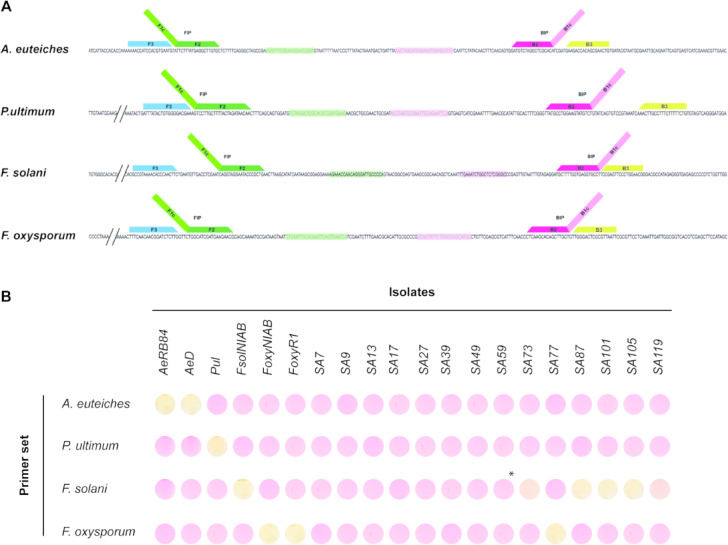
Primer design and specificity assessment. (**A**) LAMP primers were designed based on partial ITS1 sequences of the target pathogen. The Forward Inner Primer (FIP) is shown in two shades of green, with the F2 region at the 3’ end (dark green) and the F1c region in the 5’ end (light green). The Forward Outer Primer (FOP or F3) is shown in blue. The Backward Inner Primer (BIP) is depicted in two shades of magenta, with the B2 region at the 3’ end (fuchsia) and the B1c region at the 5’ end (pink). The Backward Outer Primer (BOP or B3) is shown in yellow. (**B**) Colorimetric LAMP specificity testing was conducted on a set of 20 fungal and oomycete isolates. All primer sets were tested with 10 ng of gDNA at 66 °C for 35 min, except for the *P. ultimum* primers, which required an extended incubation time of 41 min. *Isolate *SA59* turned positive after 45 min of incubation.

Consensus regions of 300–400 bp (Supplementary Fig. 2, Supplementary File 1) were identified from the alignment of multiple ITS1 sequences for each target pathogen, including *A. euteiches*,* P. ultimum*,* F. solani* and *F. oxysporum*. To test whether the ITS1 marker provides sufficient resolution to differentiate the four targeted pathogens, a phylogenetic tree was constructed using all the sequences employed in the design of the LAMP primers, along with the consensus sequences (Supplementary Fig. 3). The resulting tree showed good clustering among the different organisms tested, so the consensus regions identified were then used to design highly specific LAMP primer sets. The primers exhibited 100% query coverage and identity with the ITS1 regions of all publicly available sequence datasets for the respective pathogens in the NCBI database, with low off-target matches detected in other sequences (Supplementary Table 1). Figure 1A summarises the conserved regions and the location of the primers, while Table 2 provides the sequences of the designed primers. These primers were tested at different temperatures and incubation times and were found to be most efficient at 66 °C for 35 min using 10 ng of pure gDNA, except for the *P*. *ultimum* primers, which required an extended incubation time of 41 min.

### Specificity of LAMP assay

The root rot complex is characterised by the co-existence and co-infection of its members in both soil and plant hosts, complicating the individual detection of causal agents. Therefore, it is essential to evaluate the specificity of primer sets against a range of common fungal isolates associated with pea roots. For the specificity testing of primers, we selected different strains of *A. euteiches*, *P. ultimum*, *F. solani* and *F. oxysporum*, along with 14 additional isolates from our lab collection. These included other common pea root pathogens, such as *Phoma medicaginis* (SA7) and *Rhizoctonia solani* (SA9), as well as environmental isolates co-inhabiting pea roots, such as *Mucor* sp. and other members of the *Fusaria* group (Table 1). Given that ITS1 sequencing showed consistent clustering at species level (Supplementary Fig. 1), isolates from our lab collection were randomly selected for the specificity assays.

Following the optimized reaction conditions, we observed that the *A. euteiches* primers produced a positive yellow colour reaction exclusively with the two *A. euteiches* strains, *AeRB84* and *AeD*. The *P. ultimum* primers also yielded positive results only when paired with their corresponding gDNA, indicating high specificity for their intended targets. The *F. solani* primers successfully detected the six *F. solani* isolates, namely *FsolNIAB*,* SA59*,* SA73*, *SA87*, *SA101* and *SA105*, with a weak off-target amplification for *SA119*, previously identified as *F. redolens.* Notably, the *F. oxysporum* primers showed 100% detection efficiency, producing positive results for both the control strains (*FoxyNIAB* and *FoxyR1*) and for *SA77*, also identified as *F. oxysporum* (Fig. 1B). These results highlighted that: (i) the ITS region serves as a suitable marker for the design of species-specific LAMP primers, providing sufficient resolution to distinguish the targeted pathogens from closely related and co-occurring species, and (ii) the LAMP assay demonstrated high efficacy in detecting various members of the pea root rot complex, with cross-reactivity observed in only one isolate of a non-target species.

### Determining the detection threshold

**Fig. 2:**
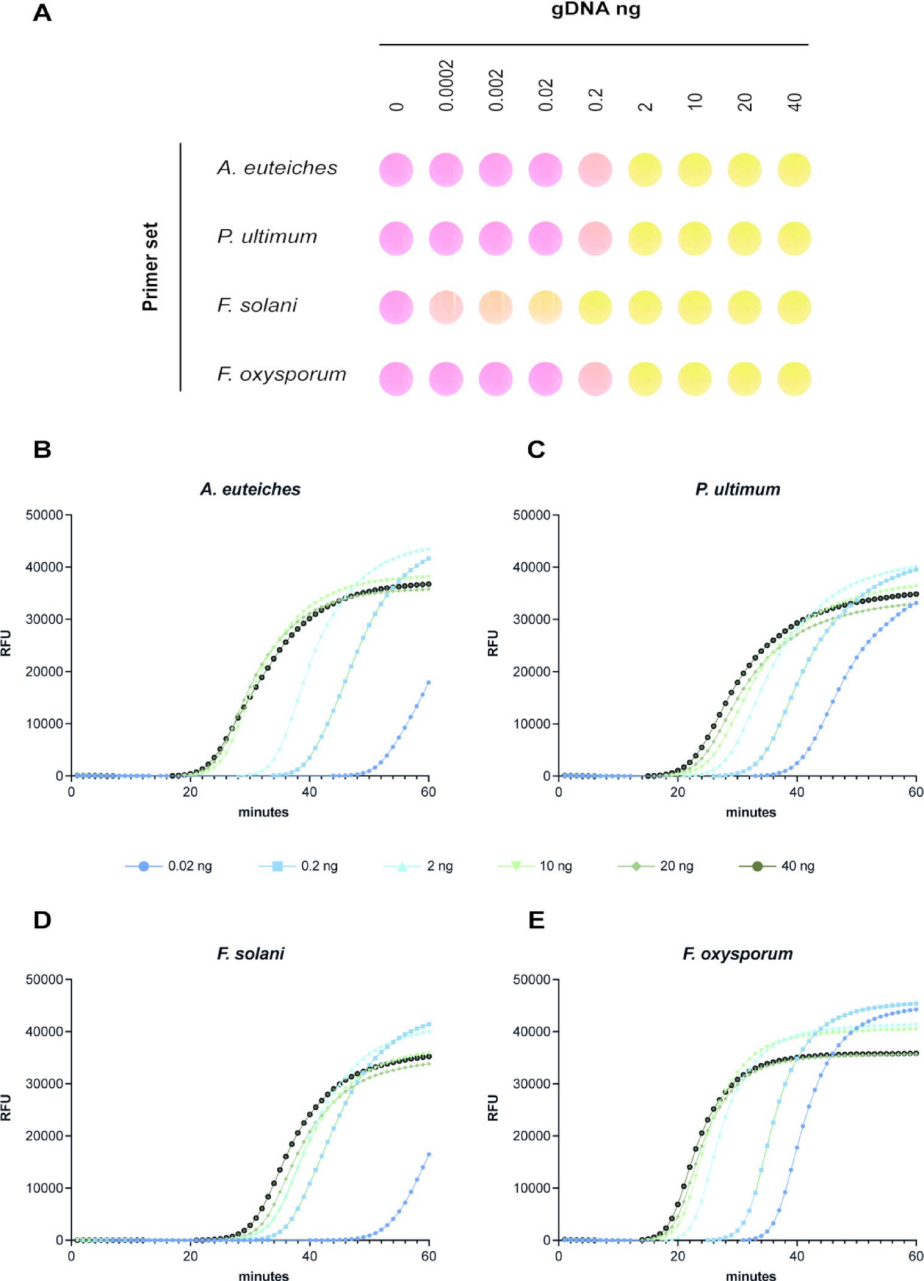
LAMP detection threshold. (**A**) Colorimetric LAMP assay conducted using four primer sets across a gradient of pure gDNA concentrations for all target pathogens. Reactions were performed at 66 °C for 35 min, except for *P. ultimum*, which required 41 min. (**B-E**) RealAmp fluorometric detection of gDNA from each pathogen using their respective primer sets. (**B**) *A. euteiches* Drechsler, (**C**) *P. ultimum* var. *ultimum*, (**D**) *F. solani* and (**E**) *F. oxysporum*. The tested gDNA concentrations in RealAamp were 0.02 ng (dark blue), 0.2 ng (light blue), 2 ng (sky blue), 10 ng (light green), 20 ng (dark green) and 40 ng (black).

To evaluate the minimum detection threshold of the targeted pathogens using the LAMP assay, a range of different gDNA concentrations was tested (Fig. 2). In the colorimetric assay, concentrations from 0.0002 to 40 ng were tested (Fig. 2A). All primer sets effectively detected their respective targets at gDNA concentrations as low as 0.2 ng under optimised reaction conditions (Fig. 2A). Notably, the *F. solani* primers showed weak but positive detection even at the lowest concentration of 0.0002 ng (0.2 pg) within 35 min. Additionally, using the RealAmp platform, concentrations ranging from 0.02 to 40 ng were also tested, with fluorescence signals observed at the lowest concentration (0.02 ng) within 40 min for all pathogens (Fig. 2B-E). At higher gDNA concentrations (≥ 10 ng), signal saturation was observed, and detection occurred as early as 20 min, particularly for *A. euteiches* (Fig. 2B). These results demonstrate that the LAMP assay is highly sensitive, reliably detecting pathogen gDNA at concentrations as low as 0.02 ng within 45 min for all the pathogens tested, except *F. solani* for which detection was achieved at 0.0002 ng within 35 min. Furthermore, detection time can be reduced to 20 min when pathogen DNA concentrations exceed 10 ng.

**Fig. 3:**
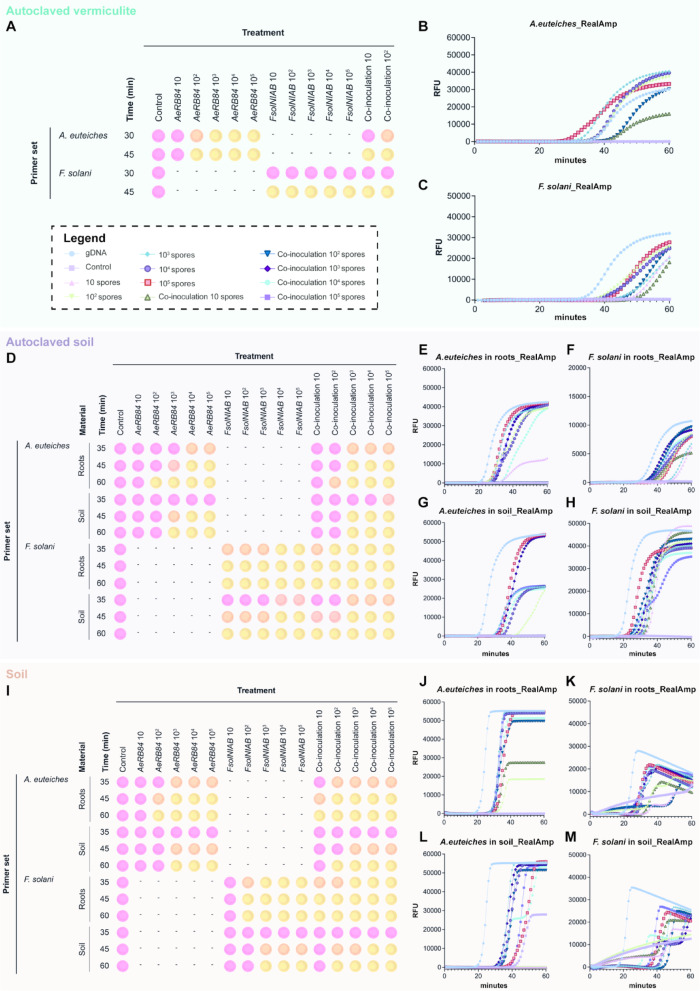
*In planta* detection by LAMP. (**A**) Colorimetric LAMP performed on root gDNA with *A. euteiches* and *F. solani* primers from plants grown in autoclaved vermiculite. The assay was evaluated at 30 and 45 min, with results based on two independent biological replicates. A representative colour change is shown here. (**B-C**) RealAmp detection of root gDNA using *A. euteiches* and *F. solani* primers from plants grown in autoclaved vermiculite. Results are based on two biological replicates and two technical replicates, with graphs showing mean values at each time point. **(D)** Colorimetric LAMP of root and soil gDNA performed with *A. euteiches* and *F. solani* primers from plants grown in autoclaved soil, evaluated at 35, 45 and 60 min. Results are based on two biological replicates, with a representative colour change shown here. (**E-H**) RealAmp detection of (E-F) root gDNA and (G, H) soil gDNA performed with *A. euteiches* and *F. solani* primers from plants grown in autoclaved soil, evaluated at 35, 45 and 60 min. Results are based on up to four biological and two technical replicates, with graphs showing mean values at each time point. (**I**) Colorimetric LAMP assay on root and soil gDNA performed with *A. euteiches* and *F. solani* primers from plants grown in pot soil for four weeks, evaluated at 35, 45 and 60 min. Results are based on two biological replicates, with a representative colour change shown here. (**J-M**) RealAmp detection of (J, K) root gDNA and (L, M) soil gDNA performed with *A. euteiches* and *F. solani* primers from plants grown in pots in standard soil. Results are based on up to four biological and two technical replicates, with graphs showing mean values at each time point. Note that for all roots samples, 20 ng of gDNA were used as input, whereas 10 ng were used in the soil samples. All concentrations referred to the initial spore concentrations.

### Evaluation of LAMP primers for *in planta* detection of *F*. *solani* and ***A***. ***euteiches*** inoculum levels

To evaluate the effectiveness of the LAMP primers *in planta*, controlled inoculation experiments were conducted using *F*. *solani* (*FsolNIAB*) and *A*. *euteiches* (*AeRB84*), both individually and as a co-inoculum. Five different concentrations of conidia or zoospores (10 to 10^5^) were used to inoculate one-week-old pea plants grown in axenic vermiculite, autoclaved soil and standard soil, to progressively evaluate the performance of our assay under different levels of biological and environmental complexity (Fig. 3). Root tissue and adjacent soil were sampled for gDNA extraction and analysis two weeks after inoculation in the autoclaved substrates and four weeks after inoculation in the standard soil.

(i) Pathogen detection using LAMP in vermiculite.

In vermiculite, colorimetric LAMP detected *A. euteiches* at an initial inoculum concentration of 10^2^ zoospores within 30 min, with partial colour change observed for co-inoculation with *F. solani*. Complete detection of all *A. euteiches* and co-inoculation concentrations was achieved within 45 min, except for the lowest concentration of 10 zoospores. For *F. solani*, all spore concentrations were detected within 45 min (Fig. 3A). No off-target amplification was observed in control plants. These results were also confirmed by RealAmp (Fig. 3B-C) which detected all *A. euteiches* concentrations above 10 zoospores within 40 min and *F. solani* concentrations, including the lowest at 10 spores, within 50 min. Notably, no fluorescence signal was observed in the control plants, even after 60 min of incubation. For initial spore concentrations between 10^3^ and 10^5^ in the co-inoculation experiment, gDNA extraction failed due to the extremely low amount of tissue recovered, likely caused by severe disease symptoms.

(ii) Pathogen detection using LAMP in autoclaved soil.

In experiments conducted with autoclaved soil in 50 ml tubes (Fig. 3D-H), the colorimetric LAMP detected *A. euteiches* concentrations at 10^4^ zoospores within 35 min and at 10^2^ zoospores after 60 min in root samples. In co-inoculation experiments with both *FsolNIAB* and *AeRB84*, detection sensitivity improved to 10^3^ zoospores at 35 min and 10^2^ zoospores by 60 min. However, using soil gDNA, the lowest concentration detected for both *AeRB84* alone and co-inoculated samples with *FsolNIAB* was 10³ spores within 60 min. For *F. solani*, all concentrations were detected in root samples within 45 min and in soil within 60 min (Fig. 3D). RealAmp analysis confirmed these findings (Fig. 3E-H). For *A. euteiches* primers, fluorescence was detected in roots within 40 min for all concentrations tested, except for co-inoculated samples with 10 and 10^2^ spores per organism (Fig. 3E). In the soil, the lowest concentration detected was 10^2^ spores for *AeRB84* alone and in co-inoculation with *FsolNIAB*, 10^3^ spores within 40 min (Fig. 3G). For *F. solani* primers, all tested concentrations were effectively detected in both roots (Fig. 3F) and the soil (Fig. 3H) in less than 50 min. As in previous tests, no signal was detected in the uninoculated control plants, indicating high specificity of the LAMP primers. These results provided an approximation of the detection threshold of our tool for assessing soil disease potential.

(iii) Pathogen detection using LAMP in standard soil.

Plants grown in pots with standard soil were evaluated to assess LAMP detection capabilities in presence of other soil microbiota (Fig. 3I-M). Colorimetric LAMP detected *A. euteiches* zoospores at 10^3^ concentrations in roots within 35 min, which improved to 10^2^ zoospores in co-inoculated samples with *F. solani*. At 60 min, the assay’s sensitivity increased, detecting 10^2^ zoospores in the single inoculations, while all tested concentrations were detected in co-inoculated samples. In soil, sensitivity was reduced by 10-fold, detecting 10^3^ spores for individual inoculation and 10^2^ spores for co-inoculation within 60 min (Fig. 3I). Using *F. solani* primers, 10^2^ spores were detected in roots within 45 min and 10^3^ spores in soil, although co-inoculation showed improved sensitivity, enabling detection of 10 spores in the roots and 10^2^ in the soil after 60 min. RealAmp confirmed these results, capturing pathogen concentrations more effectively (Fig. 3J-M). For *A. euteiches* primers in the diseased roots, we could detect concentrations above 10^2^ zoospores for *AeRB84* alone and as low as 10 spores per pathogen in the co-inoculation experiment in less than 40 min (Fig. 3J). In soil samples, initial concentrations of 10^3^ or higher were detected for single inoculation, while 10^2^ spores were detected in co-inoculated samples (Fig. 3L). For *F. solani* primers, detection was successful for all tested concentrations in both roots and soil samples within 60 min (Fig. 3K, M). Even though detection was successful, it is important to note that for *F. solani* primers, the uninoculated control in the RealAmp (Fig. 3K and M) exhibited an unexpected amplification signal. However, since the colorimetric LAMP assay (Fig. 3I) for the same uninoculated control showed no colour change even after 60 min of incubation, we are confident that this does not reflect off-target amplification, instead it is likely due to primer dimerization or secondary structure formation which can lead to non-specific intercalation of the fluorescent dye used in the RealAmp.

(iv) LAMP comparison with qPCR.

To compare the detection efficacy of the LAMP primers with qPCR, reactions were set up for all three substrates used in the experiments (Supplementary Fig. 4). In vermiculite tubes, qPCR analysis showed that *A. euteiches* detection matched LAMP sensitivity for initial inoculum concentrations as low as 10² zoospores. However, after more than 30 cycles, off-target amplification was observed for both the lowest concentration of 10 zoospores and uninoculated controls (Supplementary Fig. 4A). In the autoclaved soil, *A. euteiches* concentrations below 10^2^ zoospores could not be clearly differentiated from the uninoculated controls in both root and soil samples (Supplementary Fig. 4C, E). For *F. solani*, qPCR struggled to differentiate spore concentrations in root samples but performed better with soil samples (Supplementary Fig. 4B, D, F). In pot assays using standard soil, qPCR detected both pathogens but could not clearly differentiate between their concentrations (Supplementary Fig. 4G-J). These results conclude that our LAMP assay demonstrated equivalent or superior sensitivity compared to qPCR, with no off-target amplification.

(v) Association of LAMP results with disease severity in plants.

A clear association was observed between the LAMP detection threshold and disease severity in plants, with increasing pathogen concentrations linked to more severe symptoms (Supplementary Fig. 5). This trend was particularly evident at spore concentrations of 10^3^ or greater, especially for *A. euteiches* in microbiome-free substrates (Supplementary Fig. 5A, B). In co-inoculation assays, the synergistic effect of the pathogens further exacerbated disease severity. In standard soil, plants inoculated with pathogen concentrations of 10^4^ or above showed clear disease symptoms for both pathogens, more evident in the co-inoculation experiment (Supplementary Fig. 5C). These observations highlight the utility of LAMP for early detection and its correlation with disease progression. Samples with higher initial pathogen loads caused more severe disease symptoms, which could be reliably detected using LAMP in less than 45 min for both roots and soil.

These results conclude that colorimetric LAMP provides a rapid and qualitative tool for pathogen detection, while RealAmp has the potential to serve as an accurate quantitative alternative to qPCR for these pathogens. The high specificity of LAMP, along with its strong correlation between detection time and disease severity, reinforce its potential as an effective diagnostic tools for root rot pathogens.

### Translating LAMP to a portable diagnostic tool

**Fig. 4:**
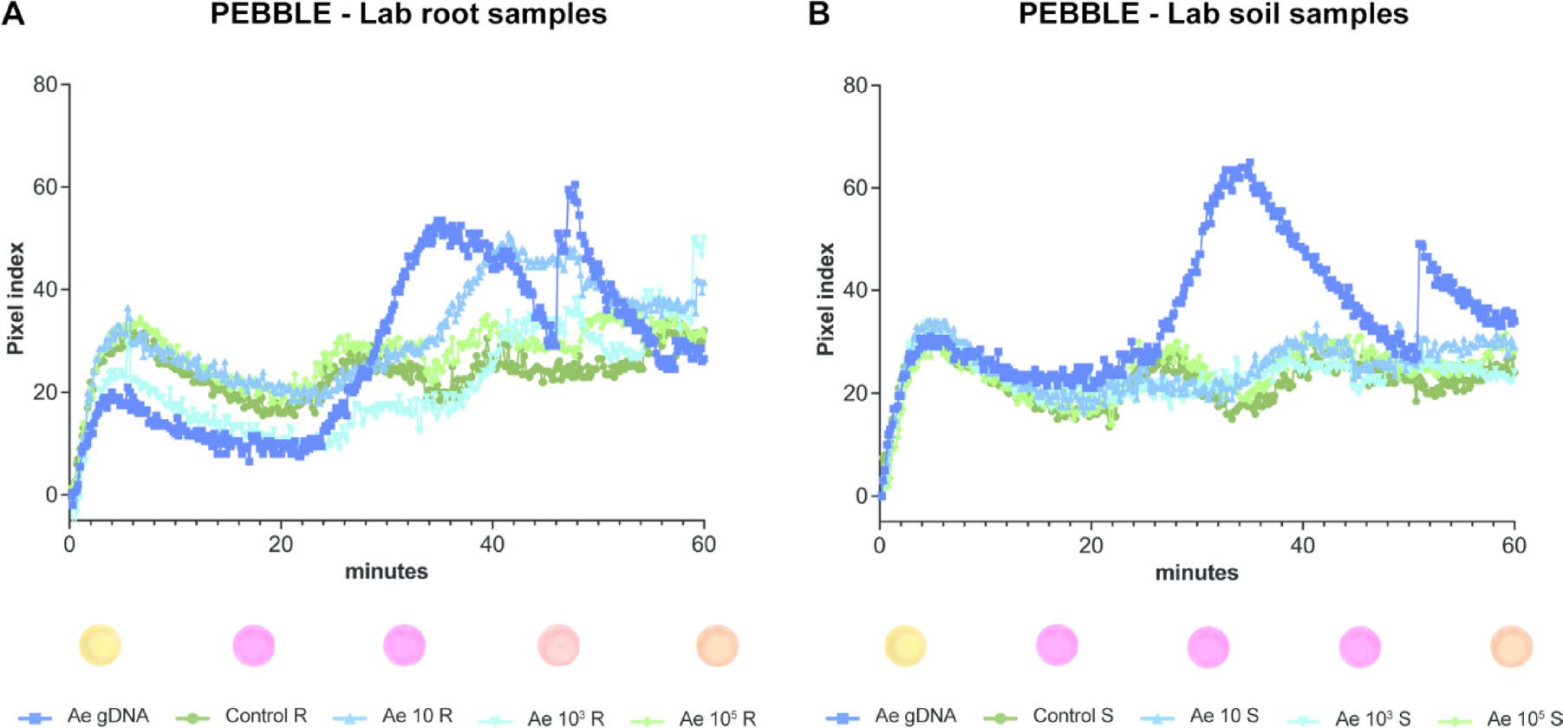
Detection of *A. euteiches* using LAMP-based portable PEBBLE device. **(A)** Results from root samples and **(B)** soil samples collected 4 weeks post inoculation. Representative images of the colorimetric change observed after 60 min of incubation in the PEBBLE device are shown below each graph. For all PEBBLE assays, gDNA from all biological replicates was pooled following extraction, and 5 µl of pooled DNA was used per reaction, with two technical replicates per sample.

The study aims to develop a diagnostics kit that is portable and suitable for use outside research facilities without the need for highly specialized technical skills. Building on advancements in portable diagnostic tools, such as the PEBBLE device by Papadakis et al.^24,25^, which enables real-time, quantitative colorimetric LAMP assays, we evaluated its applicability to our system. In this device, colour change is expressed as colour index units on the Y-axis of a real-time amplification curve and a sample is considered positive when a distinct change in the curve’s slope is observed. Initial evaluations confirmed the efficacy of our primers in amplifying different concentrations of pure gDNA for the target pathogens, with successful detection at all concentrations tested within 50 min - except for *F. solani* where 0.2 ng was the lowest detectable concentration (Supplementary Fig. 6). Following the confirmation that PEBBLE device could successfully detect the intended targets, we evaluated its suitability for detecting *A. euteiches* in 4 wpi soil samples from our previous experiment (Fig. 3I-M). These samples were processed using a fast, minimal-equipment gDNA extraction method, an adaptation of a magnetic beads-based protocol described elsewhere^23^, for subsequent analysis in the PEBBLE device (Fig. 4). The RealAmp system was used in parallel for comparison (Supplementary Fig. 7). Detection in root samples was successful for both 10^3^ and 10^5^ initial zoospores inoculum (Fig. 4A, Supplementary Fig. 7A). In soil samples, a clear positive signal was observed at the 10⁵ zoospore concentration (Fig. 4B), which was more pronounced in both PEBBLE’s reaction colour change and the RealAmp. A weak positive amplification was also detected at the 10³ inoculum level using RealAmp (Supplementary Fig. 7B).

**Fig. 5:**
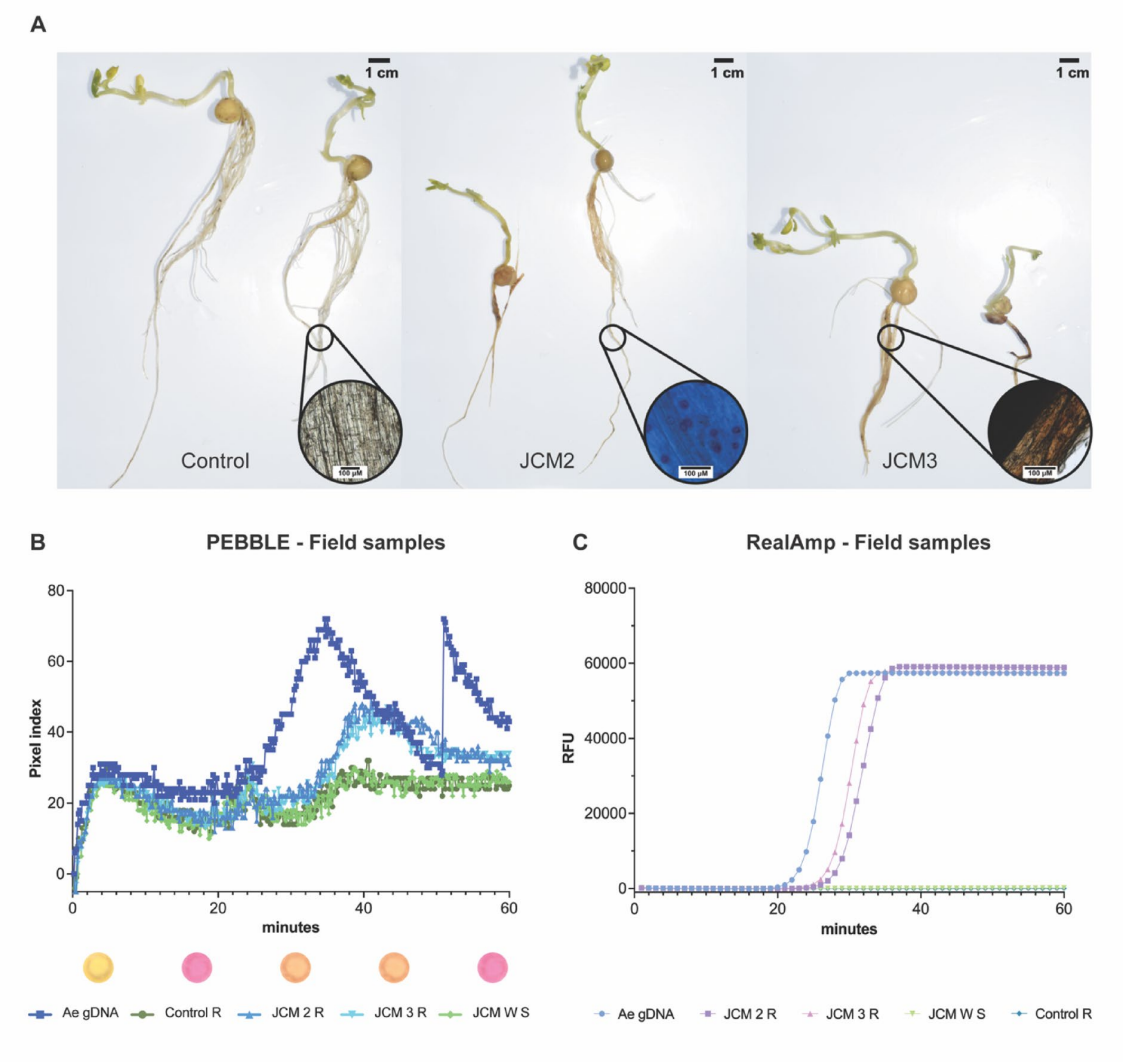
Validation of PEBBLE using field samples. **(A)** Soil baiting assay of 10 days old plants using agricultural field soil. Zoom-in microscope images taken at 40X. Images show a representative example from all the samples assessed. **(B)** Field samples PEBBLE results. **(C)** Field samples RealAmp results. Biological replicates were pooled together following gDNA extraction and 5 µl were used per reaction in two technical replicates.

### Validation of PEBBLE using field samples

After demonstrating that the portable PEBBLE device, in combination with the magnetic bead-based extraction, produced results comparable in accuracy to those observed in the lab experiment, we extended the assay to field samples from sites with known history of pea root rot. Given that in the UK soil baiting with pea seedlings is used as the standard diagnostic method for detecting root rot pathogens^22^, we first set up baiting plates using pea seedlings and field-collected soil samples. Of the 21 soil samples tested, oospores (a definitive sign of *A. euteiches* infection) were observed in only one sample (JCM 2 R), while 13 plates exhibited characteristic honey-brown discoloration. A summary of these phenotypes is presented in Fig. 5A. Control plants displayed healthy roots, while infected samples (JCM 2 R and JCM 3 R) showed honey-brown discoloration and dark lesions typical of *Fusarium* infections^6^. Notably, honey-browning often co-occurred with darker lesions even in the absence of visible oospores (Fig. 5A, Supplementary Table 2).

We extracted gDNA from these samples using both a column-based method and our magnetic bead protocol, followed by analysis with the PEBBLE and RealAmp platforms. Diseased root tissues from the field experiment tested positive for *A. euteiches* (JCM 2 R and JCM 3 R), while the bulk mixed soil (JCM W S) and control root samples yielded negative results (Fig. 5B and C; Supplementary Fig. 8). In all cases, no positive signal was detected in the control samples, confirming the high specificity of the assay towards the target pathogens. Altogether, the positive results obtained with the PEBBLE device coupled with the quick gDNA extraction method open an exciting opportunity for a more accessible pea root rot test, delivering results in as little as 60 min.

## Discussion

The root rot complex, caused by soil borne pathogens, presents a significant challenge to pea cultivation, limiting both yield and the potential expansion of the crop due to high disease pressure^26^. Affected fields often display large patches of yellow, stunted plants with poorly developed root system, and severe infection can result in complete yield loss^27^. To address this challenge, effective management strategies are crucial to mitigate the economic impact on farmers. These include implementing chemical and biological control measures, adopting various cultural practices and efforts to identify genetic sources of resistance. However, no effective fungicides or fully resistant pea cultivars are currently available. This challenge is compounded by the persistence of primary inoculum as dormant spores in the soil for over a decade, leading to pathogen build up across cropping cycles. In regions such as France, the USA and Canada, inoculum levels have risen so high in some pea growing regions that production and processing facilities have been relocated to less infected areas. In the UK, vining peas for the frozen pea market are restricted to a small area on the east coast due to climatic conditions and the requirement to grow them within 150 min of processing factories. This creates significant pressure on available land, contributing to declining pea yields^28^.

A key strategy for mitigating root rot associated yield losses is for growers to assess the soil’s inoculum potential before planting and select fields with low disease levels, enabling a precise and well-informed sowing plan^28,29^. Traditionally, diagnosing the pea root rot complex has involved assessing the disease severity in the field using a scale to evaluate root discoloration and visual symptoms on the plants. This is followed by determining the root rot incidence, defined as the proportion of affected plants relative to the total number sampled^30,31^. However, this method does not identify the causal agent(s) of the disease. Identifying the pathogens responsible requires specialised laboratory techniques, such as microscopic tissue examination, pathogen isolation and DNA sequencing^32,33^. Isolating members of the root rot complex from infected soil is often challenging and typically requires an additional soil baiting step to ensure root infection. In this process, a susceptible pea cultivar is planted into the suspected diseased soil under environmental conditions optimised for disease manifestation. The plants are then assessed for disease severity, and the inoculum potential of the soil is evaluated. Tissue samples are subsequently plated onto selective media for pathogen characterisation^34–37^. While these techniques are quite reliable, they are time-consuming and costly, with results often taking between 2 and 6 weeks to obtain^22^.

To address these limitations, molecular detection methods, particularly PCR-based techniques, have advanced significantly for detecting root rot pathogens. They methods circumvent the challenges of culturing certain pathogens while also allowing for precise pathogen quantification using quantitative PCR (qPCR)^38^. Since 2002, qPCR has become a key tool for quantifying *A. euteiches*, initially in alfalfa and later adapted for pea and other legumes^39^. 13 and colleagues further improved its sensitivity, enabling detection of very low concentrations, as few as 10 oospores per gram of soil^12^. In 2018, specific primers were developed for *A. euteiches*, *F. solani*, *F. avenaceum* and *F. redolens*^13^, facilitating studies on their interactions and impact on disease severity. These studies suggest that pea plants infected with *A. euteiches* are more susceptible to *Fusarium* root rot, potentially exacerbating yield loss in affected areas^6^.

**Fig. 6:**
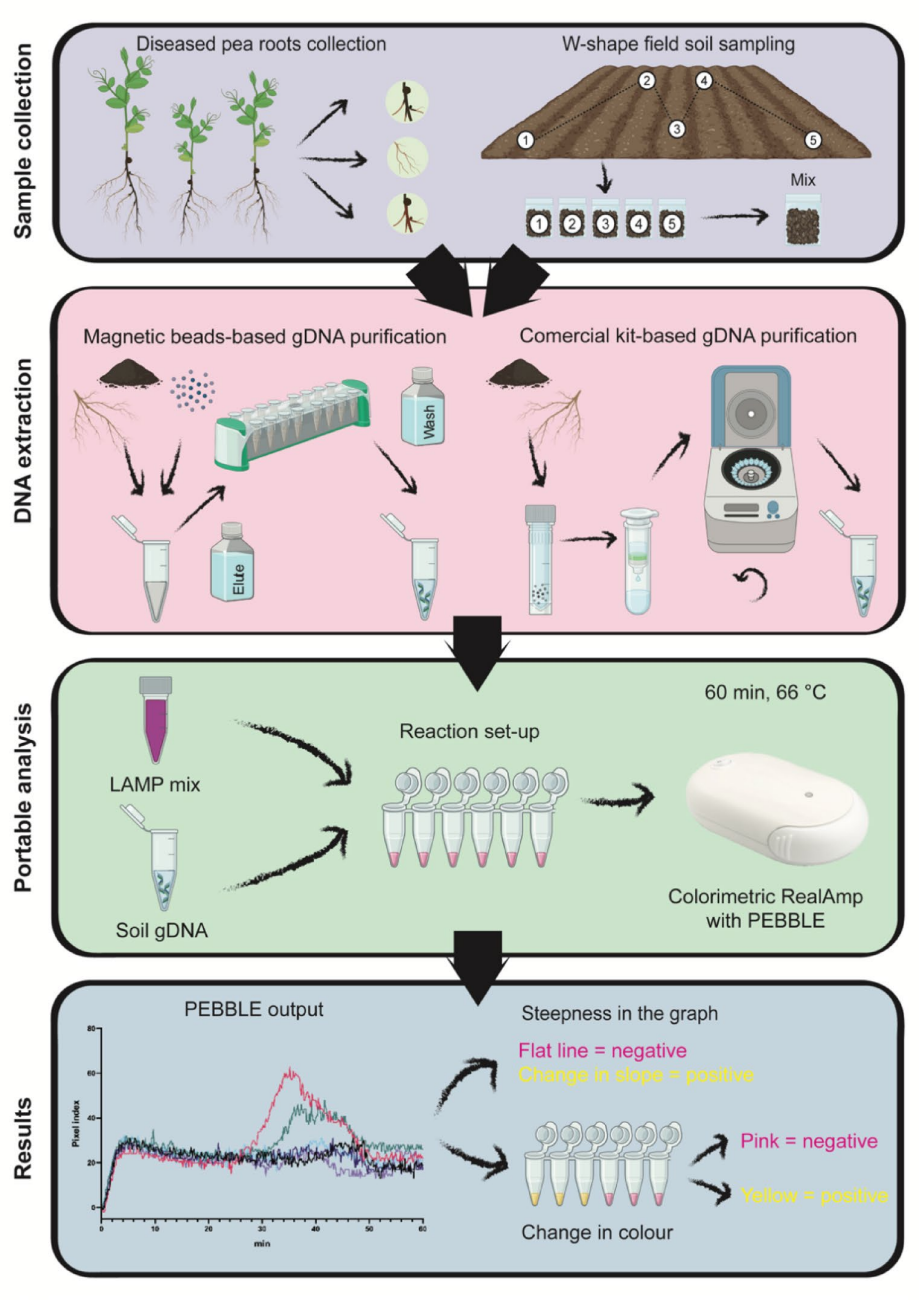
Proposed workflow for detecting root rot pathogens using the portable PEBBLE device. Samples can be collected from either diseased roots or field soil following a traditional W-shape sampling pattern, followed by soil homogenisation. Sample gDNA extraction can be performed using a commercial column-based method if laboratory facilities are available or using a magnetic bead method for rapid, field-compatible isolation. The extracted gDNA is combined with a LAMP mix containing all necessary reagents for the reaction, by subsequent incubation in the PEBBLE device for 60 min. After incubation, results can be read on the PEBBLE smartphone app or visually by observing a colour change in the reaction tube.

Additionally, in recent years, droplet digital PCR (ddPCR) has been extensively used as a risk assessment tool in France and Canada due to its ability to detect rare DNA targets in complex samples such as the soil. This method offers enhanced quantification accuracy and greater resistance to PCR inhibitors, making it a promising choice for quantifying low microbial inocula in soil^14,40^. While PCR-based diagnostics, including ddPCR, are accurate for predicting disease levels in the soil, they require a specialised laboratory setup and highly trained personnel for careful sample preparation and optimisation. This significantly increases the cost limiting their widespread adoption by growers. To improve accessibility and reduce costs, there is a growing interest in developing portable diagnostic platforms and simplified workflows for field-based pathogen detection.

In this study, we developed a rapid and cost-effective diagnostic tool leveraging LAMP technology, with the potential to be adapted into a portable diagnostic kit for field-based testing. The assay targets a universal marker, the Internal Transcribed Spacer (ITS) region, to detect four key members of the pea root rot complex, *A. euteiches*, *P. ultimum*,* F. solani* and *F. oxysporum.* These pathogens were targeted in our LAMP assay based on crop clinic report published by PGRO as well as expert technical advice obtained from pea growers and agronomists in the UK^41^. We first evaluated the assay specificity against closely related species and other common fungal members of the pea root microbiome to ensure accurate targeting of the intended pathogens. Its sensitivity was then tested to determine the lowest detectable levels of target DNA. The assay was further assessed for its practical application through *in planta* and soil-based detection, focussing specifically on the most problematic pathogens in the UK, *F. solani* and *A. euteiches*^42^. Finally, a portable detection device integrated with a fast gDNA extraction method was implemented for on-site diagnosis of root rot (Fig. 6).

Primer design is the first critical step in developing any molecular-based detection tool, particularly for LAMP, where the formation of secondary dumbbell-like structures is essential for efficient target amplification. For our study, we selected ITS1 as the target region (Fig. 1A) due to its combination of highly conserved and highly variable sequences. ITS1 is also present in multiple copies across all organisms which enhances detection sensitivity^43,44^. Although other molecular markers, such as the translation elongation factor 1-alpha (EF-1α) gene, can provide higher taxonomical resolution particularly among closely related species, EF-1α is typically present as a single copy which may reduce detection sensitivity at low pathogen concentrations^45^. However, ITS multicopy nature can complicate precise quantification, especially at low concentrations. For example, in *A. euteiches*, significant variation in ribosomal gene (rDNA) copy number has been reported among isolates, influenced by factors such as geographical origin. To address this variability, average rDNA copy numbers have been established, ranging from 95 in haploid cells to 190 in diploid cells^12,14^. Although the nature of LAMP-amplified products does not enable direct correlation with gene copy number, the formation of multi-sized stem-loop structures during LAMP serves as the starting point for subsequent exponential amplification, enabling rapid and highly sensitive pathogen detection^46^.

After testing the in silico specificity of primers, we evaluated their specificity against a diverse set of 18 fungal and two oomycete isolates (Fig. 1B). Our primers demonstrated high specificity, with one exception, the *F. solani* primer set which showed weak off-target amplification with SA119, a *F. redolens* isolate. This result was not entirely unexpected, as these species are closely related, and our reference *F. solani* isolate, *FsolNIAB*, and SA119 share about 84% sequence identity, which increases to up 100% in the binding region of the FIP primer. Our results highlight the challenges and limitations of achieving precise detection among members of the same genus when relying on a single marker which emphasise the need to expand publicly accessible genomic databases. Such databases would facilitate the design of unique primers based on species or isolate specific genomic motifs, significantly improving detection accuracy. This approach has been already exploited for several bacterial species, such as *Pectobacterium parmentieri* in potato, as well as foodborne pathogens including *Salmonella*, *Vibrio*, *Staphylococcus* and *Escherichia*^47,48^. Furthermore, the development of novel software tools like Genome based LAMP primer designer (GLAPD) has revolutionised primer design by utilising whole genomes instead of specific gene regions. This innovation enhances the success rate of designing primer sets for identifying specific organisms and accelerates the adoption of LAMP technology across various fields such as food quarantine, agricultural diagnostics and epidemic disease surveillance^48^.

Determining the threshold of detection is another critical step in developing any diagnostic tool. To achieve this, we tested a range of different gDNA concentrations of the root rot pathogens using both colorimetric and fluorescence-based LAMP assays (Fig. 2). For the colorimetric LAMP assay, gDNA concentrations ranging from 0.0002 to 40 ng were tested. A concentration of 0.02 ng was the lowest reliably detected for all pathogens, except with the *F. solani* primers, which produced a visible colour change even at 0.0002 ng (0.2 pg). Using RealAmp, detection sensitivity was evaluated for concentrations ranging 0.02 to 40 ng. All primer sets successfully detected target DNA at 0.02 ng (20 pg) within 45 min, demonstrating its potential as a rapid and sensitive diagnostic tool for identifying root rot pathogens. In studies of other phytopathogenic fungi, the detection limit of LAMP-based systems varies widely. For instance, in the case of *Talaromyces flavus*, a soilborne fungus associated with contamination of heat-processed food, the detection limit was as low as 1 fg of gDNA. In contrast, for the rice pathogen *Magnaporthe oryzae*, the detection limit was 10 pg of gDNA, achieved within a 45-minute incubation period^49,50^. This variability in detection limits emphasises the necessity of establishing calibration assays specific to each individual system, as factors such as the characteristics of the targeted sequence, primer efficiency and the type of the detection method employed can all influence performance^46^. Additionally, the inclusion of loop primers in the primer mix is known to improve detection time by up 50%, as they increase the number of initiation sites during the reaction^51^. However, in our study, the regions identified as unique to each pathogen did not allow the design of loop primers, likely due to insufficient physical space between the outer and the inner primer binding sites.

A major challenge in root rot diagnostics for pea lies in establishing a clear correlation between pathogen load and disease severity. In this study, we conducted disease severity assays coupled with LAMP detection for two major pea root rot pathogens, *A. euteiches* and *F. solani*, with the aim to determine initial inoculum load required to produce visible disease symptoms and assess whether our method could effectively detect this threshold (Fig. 3, Supplementary Fig. 5). We opted for this approach because following inoculation, fungal and oomycete biomass likely increased, potentially including both mycelial mass and resistant structures such as oospores, making direct correlations with spore or zoospore numbers technically challenging and less precise when using LAMP. We also evaluated three different growth substrates to assess the effect of soil characteristics and the soil microbiome on both detection and disease severity. Under axenic conditions, such as vermiculite or autoclaved soil, the sensitivity of the LAMP assay was significantly higher compared to standard soil, particularly when detecting pathogens in soil samples. For *F. solani*, as few as 10 spores in the initial inoculum were reliably detected in both axenic conditions, regardless of whether the input material was soil or roots. However, this detection limit decreased by 10 to 100-fold when standard commercial soil was used for roots or soil analysis, respectively. Visual inspection of the plants clearly showed disease symptoms even at lower concentrations, though the symptoms were less severe in soil than those observed in axenic conditions (Supplementary Fig. 5). This suggests a potential role of the soil microbiota in modulating disease response. The rhizosphere microbial community benefits the plant host in various ways, such as by promoting plant growth, protecting against pathogens, improving nutrient acquisition or conferring adaptive advantages. However, pathogen invasion is known to disturb this equilibrium, leading to shifts in microbial community structure^52-54^. In pea root rot, this phenomenon has not been extensively studied, though a few studies have begun to shed light on the interactions between the plant, pathogens, and their microbial community. For instance, a study by Wille et al., showed that diseased roots in infested agricultural fields were dominated by *F. solani* and *A. euteiches*, surpassing their normal fungal root community^4^. Another study showed that bacterial root communities are more affected by plant health status than fungal communities. Healthy plants exhibited a higher relative abundance of *Rhizobium*, *Olpidium* and *Morteriella*, but lower abundance of *Pythium* and *Fusarium.* The influence of these microbial shifts on diagnostic accuracy has been discussed in studies employing qPCR or ddPCR techniques. These studies highlight the potential impact of microbial consortia on disease establishment and emphasised the need for further research to fully understand the dynamics of root rot complex and its interaction with the microbiome^12,14^.

The concept of point-of-care testing has been extensively studied in human diseases^55^, with a notable example during COVID-19 pandemic when several in situ technologies were rapidly developed and implemented. These advancements demonstrated that widespread testing was essential for containing the virus^56^. For plant diseases, particularly within the European Union, pathogens and pests detection techniques are regulated by the European and Mediterranean Plant Protection Organization (EPPO)^57^. These regulations often required multiple validated assays for each organism, which can be time-consuming, costly and dependent on highly trained personnel^58^. Therefore, the implementation of alternative diagnostic methods are needed to accelerate the detection process and improve response times. With this goal, we integrated existing technologies and developed a rapid assay for detecting root rot pathogens, designed to be easily translatable to field conditions. Our method demonstrated the capability to detect *A. euteiches* at concentrations that caused visible disease symptoms both *in planta* and in soil, strengthening the effectiveness of our method. To maximise the utility of our tool, future studies should include a broader range of *in planta* assays with additional members from the root rot complex, large-scale surveys using agricultural soil with different physicochemical properties and pathogen loads.

We propose here a LAMP-based molecular diagnostics tool for the independent detection of four key pathogens in the root rot complex, with the potential to be integrated into a fully portable workflow (Fig. 6). Both soil and root samples can serve as the input material to be subjected to different gDNA extraction methods based on the available resources and expertise. A traditional column-based gDNA extraction method provides higher yields but requires a laboratory set-up, increasing the cost per reaction to £8.97. While the optimised magnetic beads-based extraction method can be performed in a non-specialised environment yielding sufficient DNA for detection at a lower cost of £3.83. After the extraction, the reaction can be performed using the PEBBLE device which accommodates up to six samples simultaneously. The total cost of this six-sample assay is £53.79 with the column-based extraction and £ 23.00 with the magnetic bead extraction. The addition of an internal positive control using pure gDNA is advisable to ensure proper reaction development. The samples are incubated for an hour and real-time results are displayed through a smartphone app. While the app predicts positive or negative results based on slope change, users can export the data for further analysis. At the end of the run, a colour change provides additional confirmation. Negative samples remain pink while positive samples turn yellow, and shades of orange indicate a low-level presence of the target organism.

With this work, we aim to contribute to the expansion of management strategies for pea root rot complex by enabling (i) accurate and timely diagnosis (ii) proactive decision-making before disease onset (iii) reduction of the associated risks to the crop, and ultimately, a decrease in costs for growers. Moreover, the flexibility of this technology allows for rapid adaptation to detect new pathogens and emerging threats by targeting precise genomic regions, making it a versatile addition to the toolkit for crop disease management.

## Supplementary Information

Below is the link to the electronic supplementary material.

Supplementary Material 1

Supplementary Material 2

Supplementary Material 3

Supplementary Material 4

Supplementary Material 5

Supplementary Material 6

Supplementary Material 7

Supplementary Material 8

Supplementary Material 9

Supplementary Material 10

Supplementary Material 11

Supplementary Material 12

## Data Availability

No datasets were generated or analysed during the current study.
