# Supplementary figures and images for: Loop mediated isothermal amplification (LAMP) as a rapid and portable diagnostic tool for the detection of pea root rot pathogens
Source: Sci Rep. 2025 Oct 7;15:34904. doi: 10.1038/s41598-025-18738-9 (PMC12504703; doi:10.1038/s41598-025-18738-9)

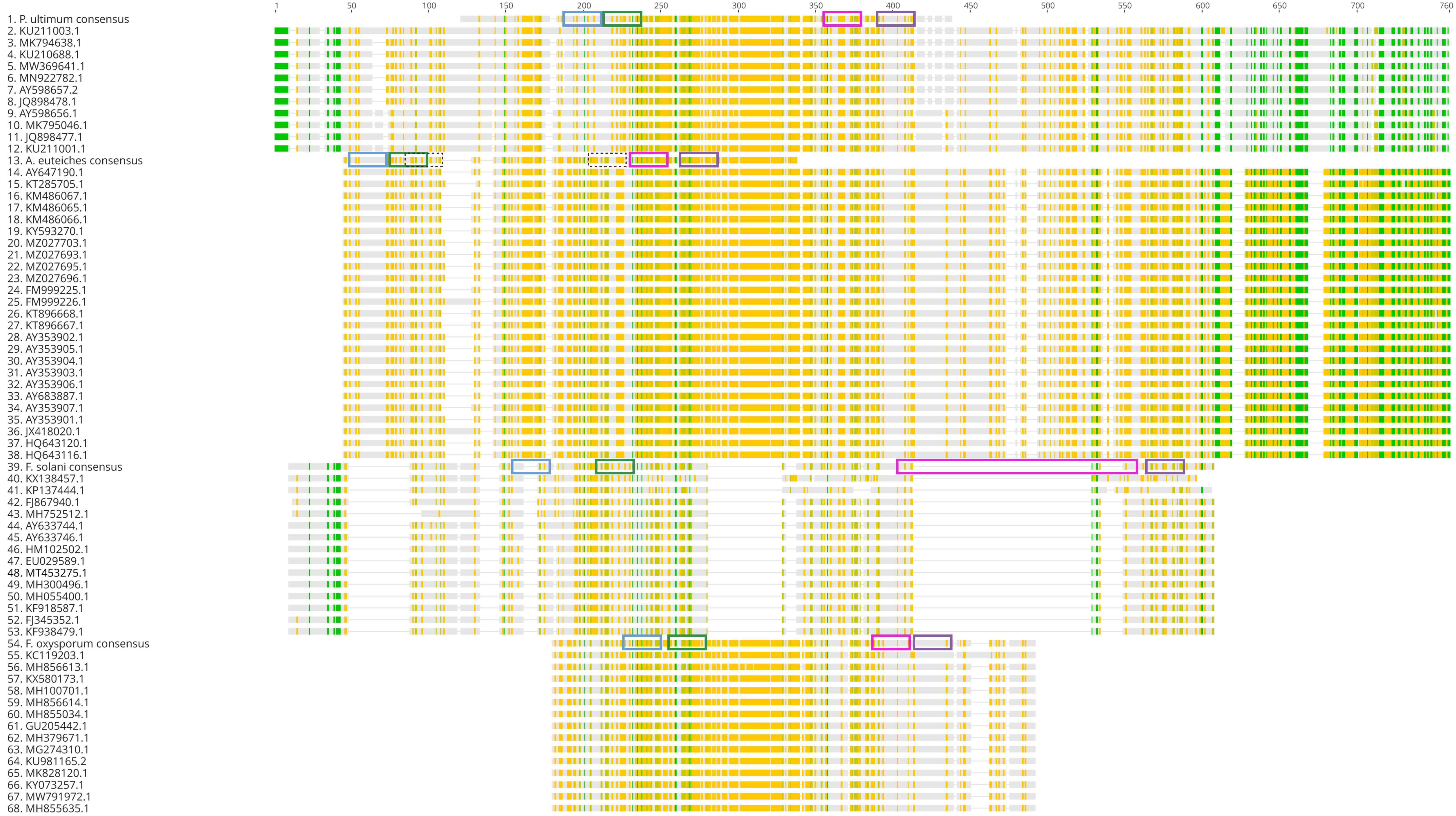

Supplement: Supplementary file 3 — Supplementary Material 3 [file 41598_2025_18738_MOESM3_ESM.tif]

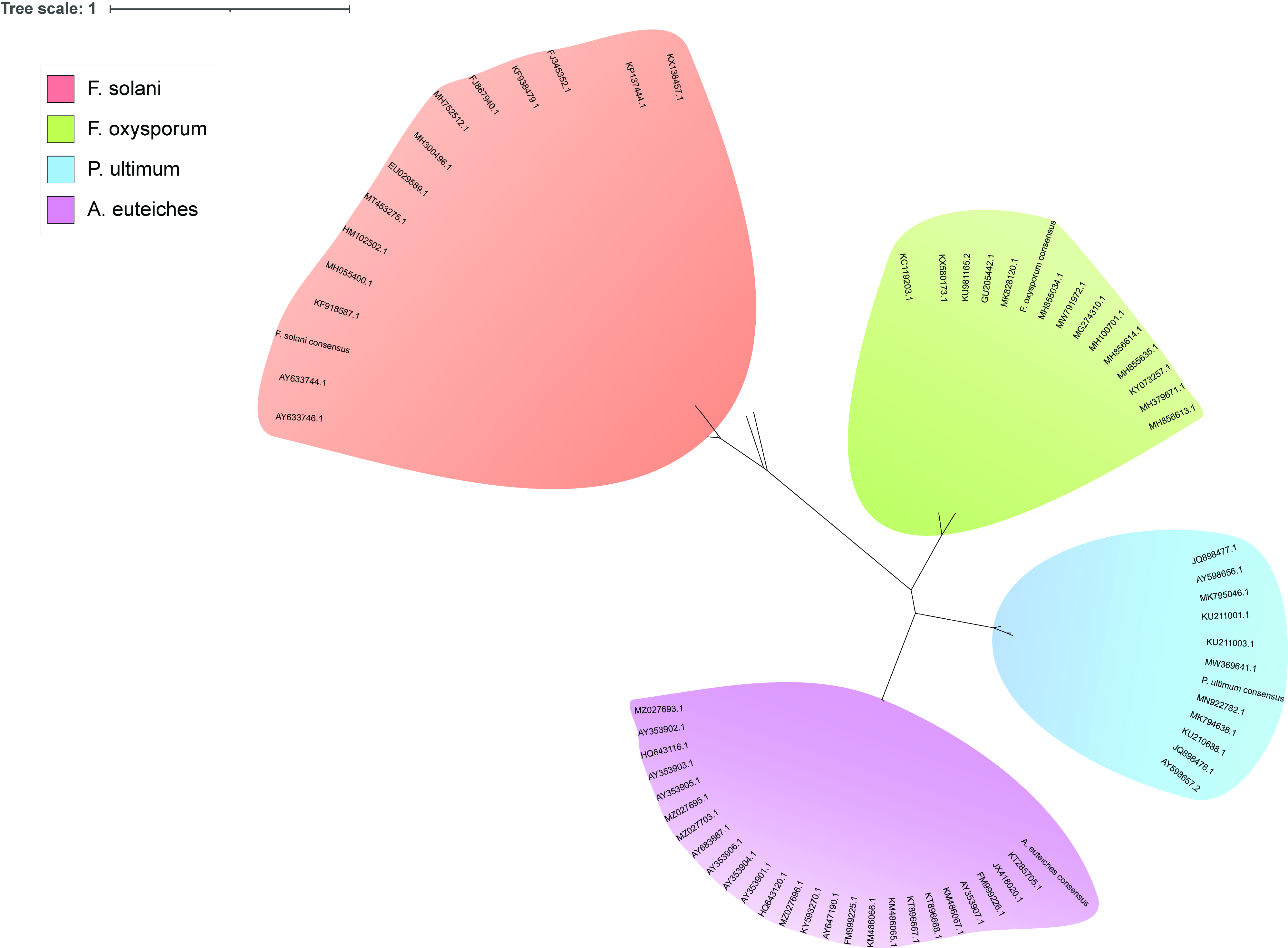

Supplement: Supplementary file 4 — Supplementary Material 4 [file 41598_2025_18738_MOESM4_ESM.tif]

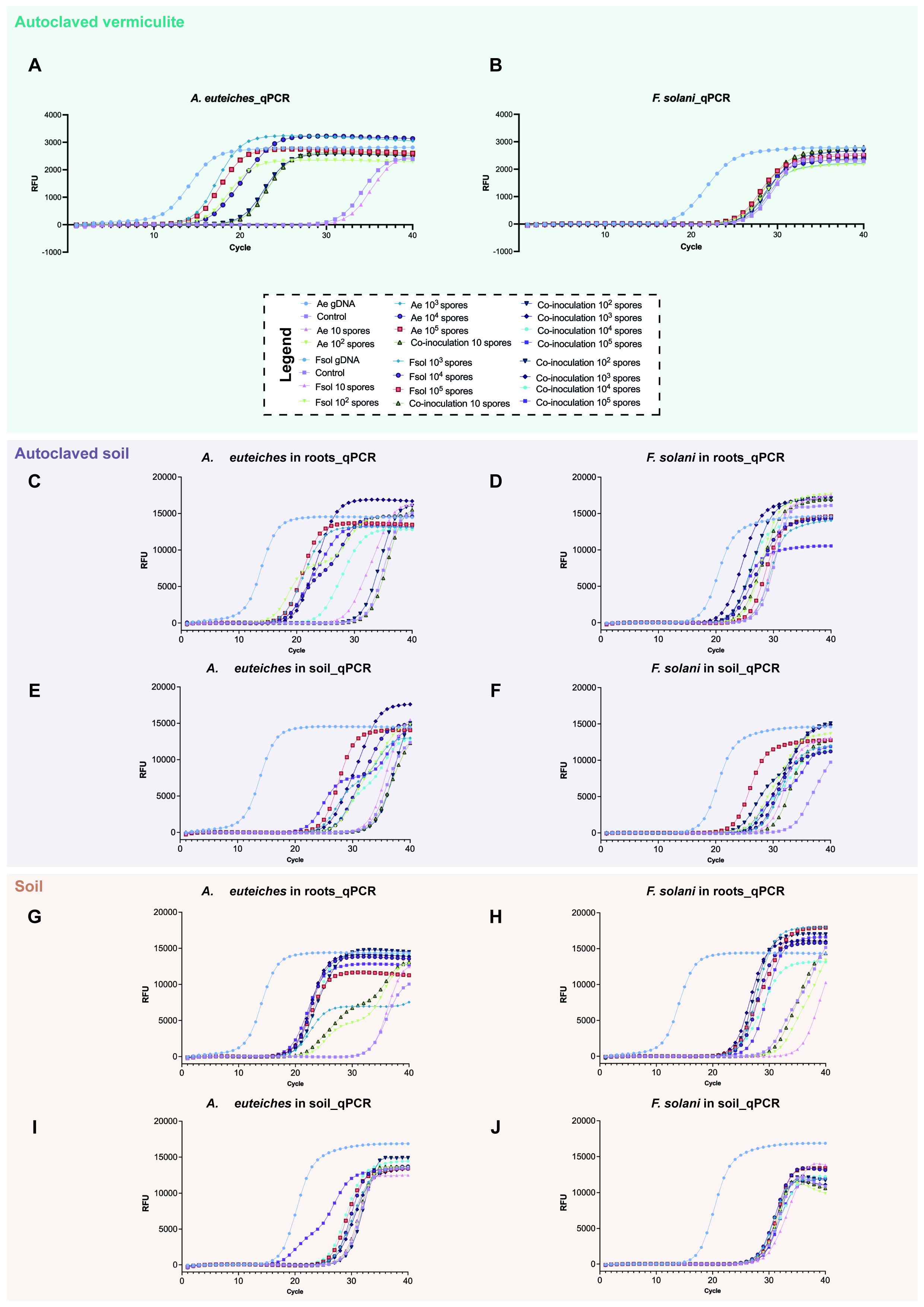

Supplement: Supplementary file 5 — Supplementary Material 5 [file 41598_2025_18738_MOESM5_ESM.tif]

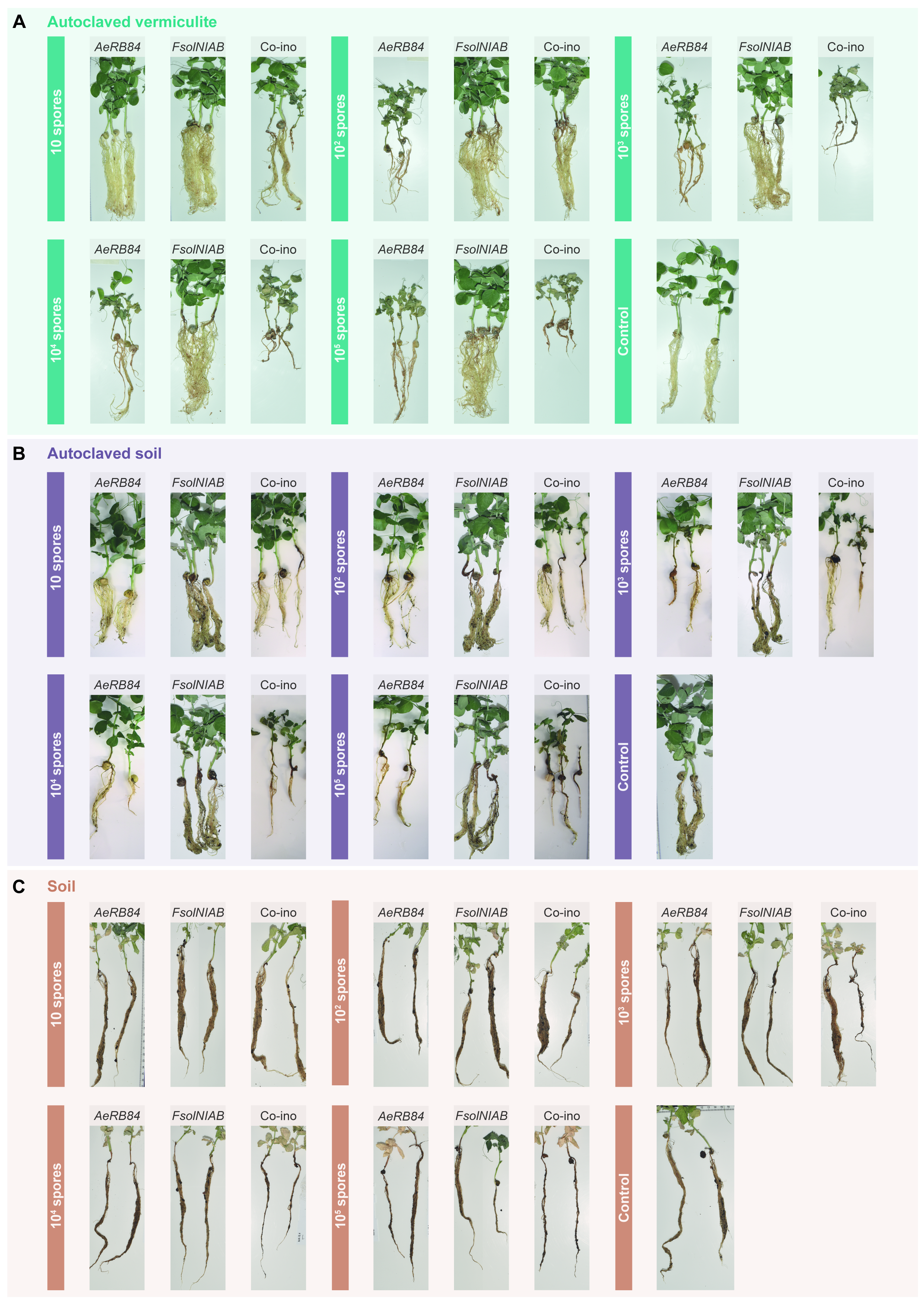

Supplement: Supplementary file 6 — Supplementary Material 6 [file 41598_2025_18738_MOESM6_ESM.tif]

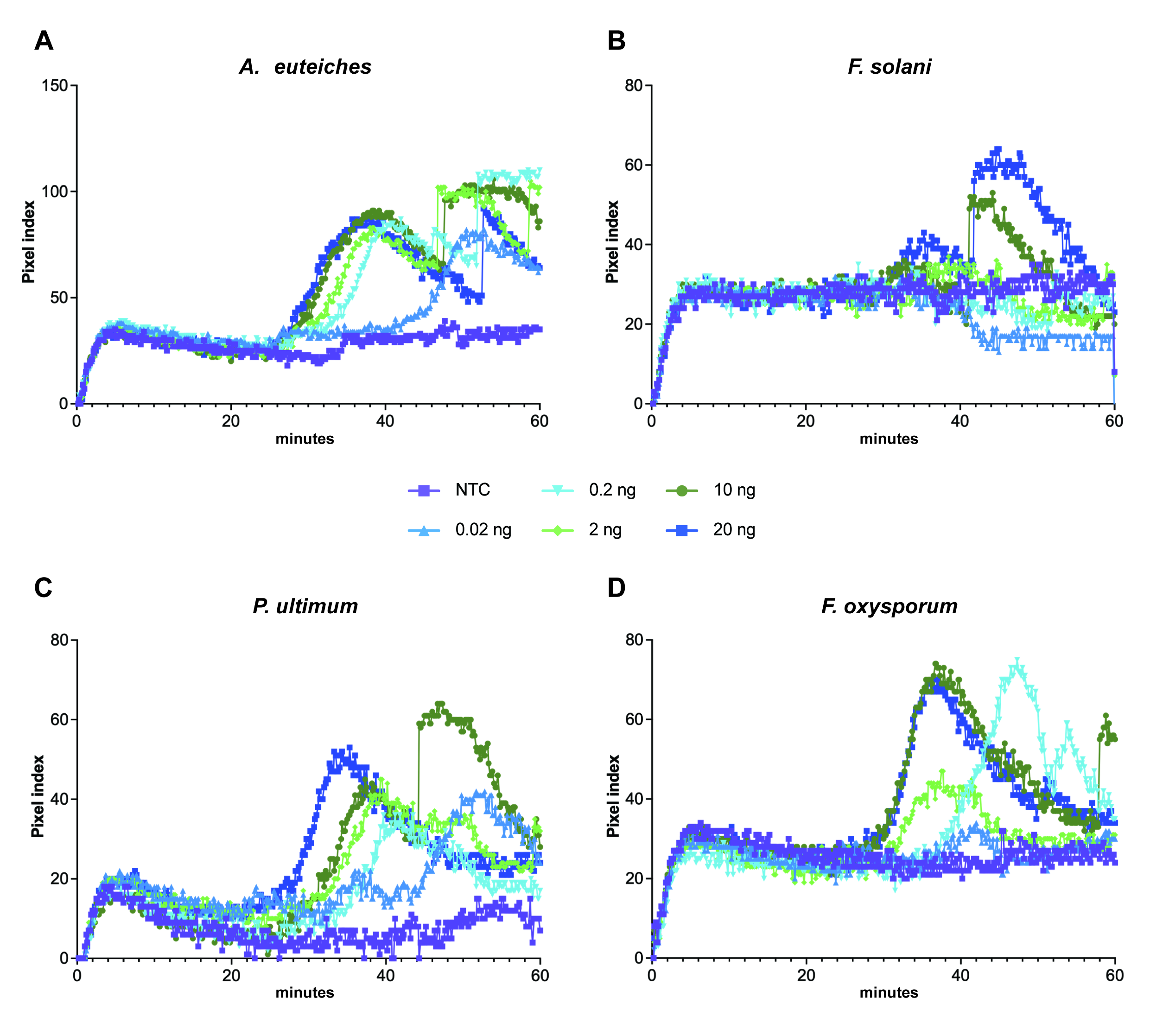

Supplement: Supplementary file 7 — Supplementary Material 7 [file 41598_2025_18738_MOESM7_ESM.tif]

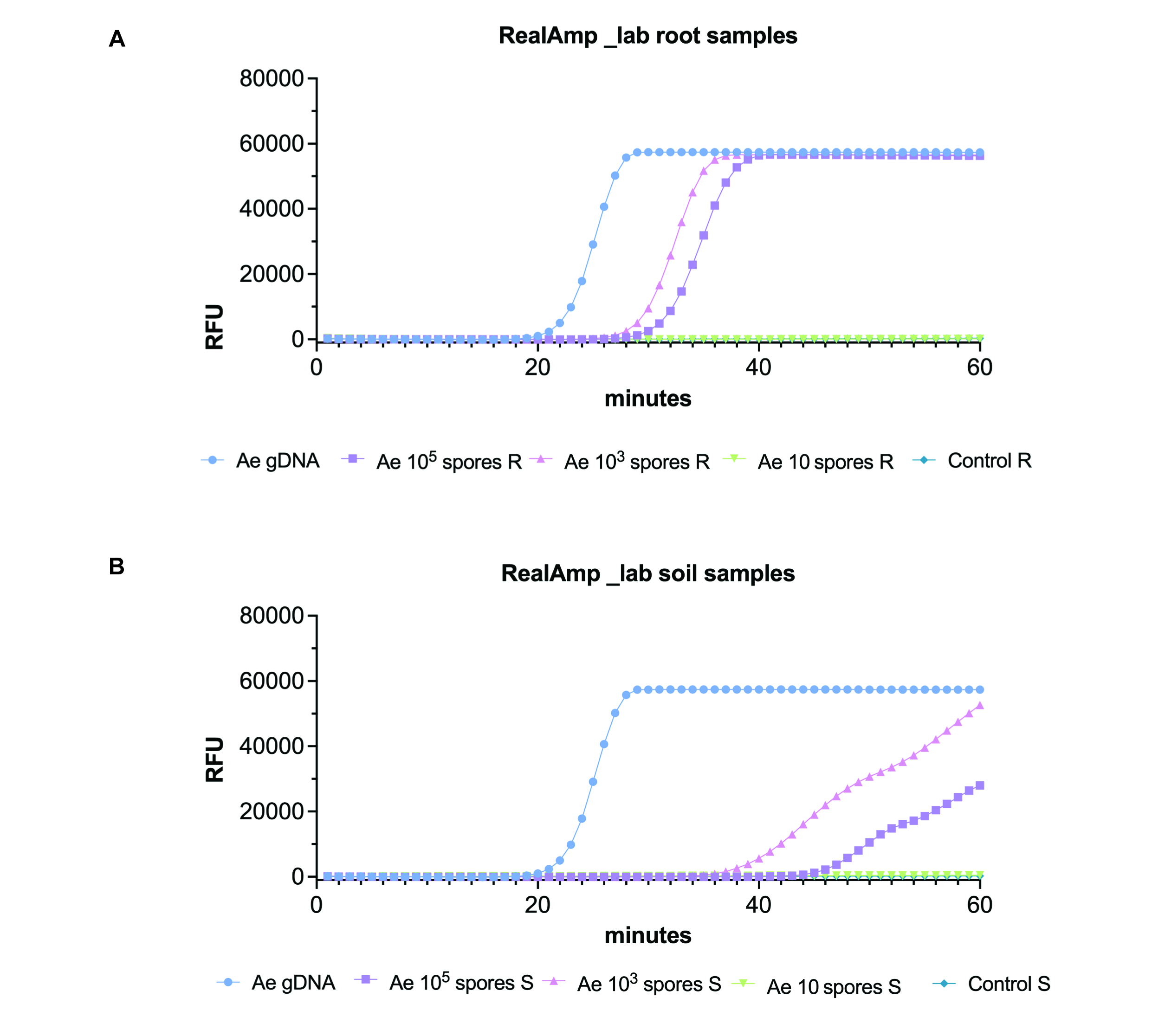

Supplement: Supplementary file 8 — Supplementary Material 8 [file 41598_2025_18738_MOESM8_ESM.tif]

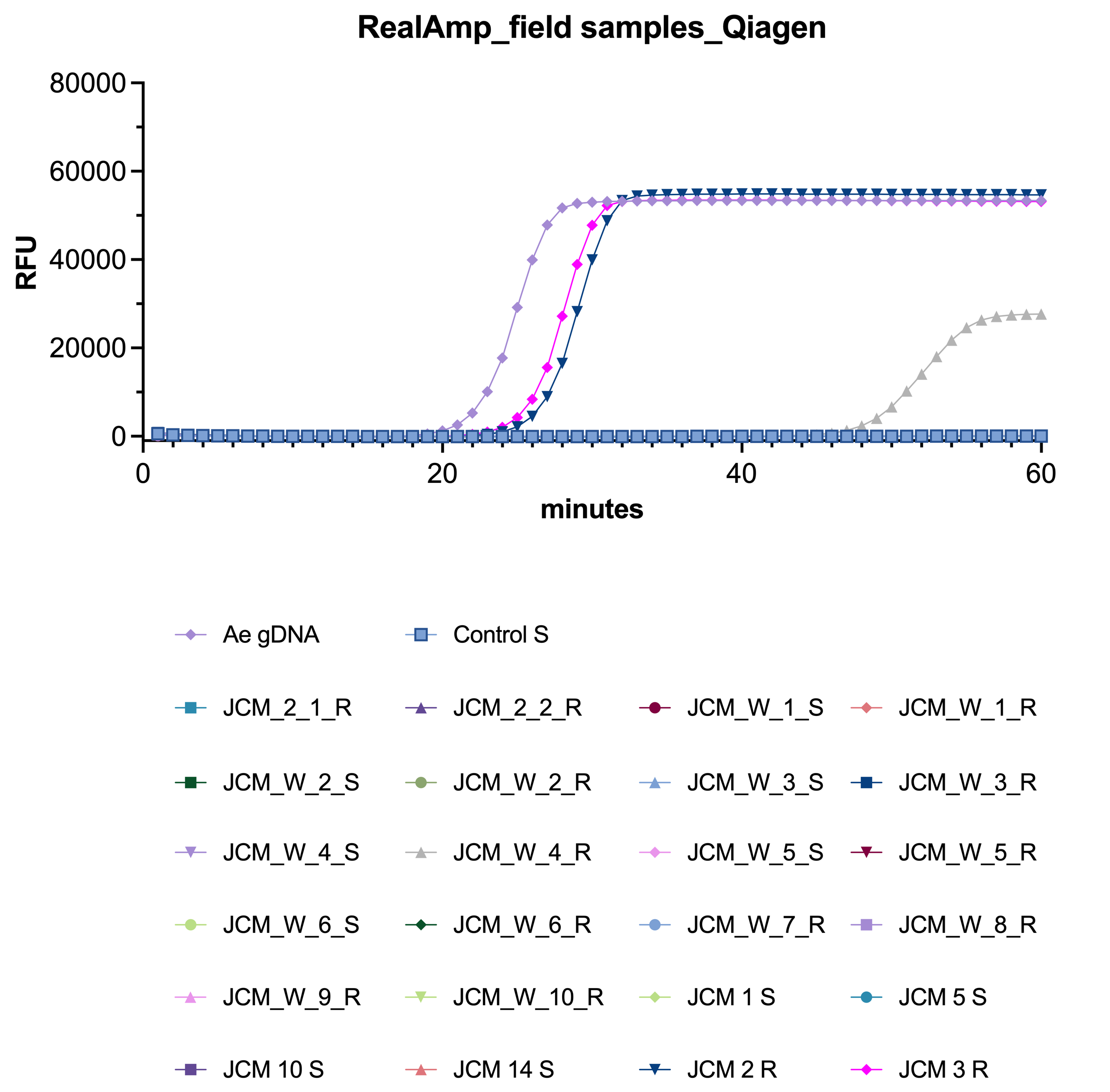

Supplement: Supplementary file 9 — Supplementary Material 9 [file 41598_2025_18738_MOESM9_ESM.tiff]
