# Supplementary material for: Loop mediated isothermal amplification (LAMP) as a rapid and portable diagnostic tool for the detection of pea root rot pathogens
Source: Sci Rep. 2025 Oct 7;15:34904. doi: 10.1038/s41598-025-18738-9 (PMC12504703; doi:10.1038/s41598-025-18738-9)
Supplement: Supplementary file 12 — Supplementary Material 12 [file 41598_2025_18738_MOESM12_ESM.docx]

**Supplementary Information**

**Supplementary Figure 1. ITS1-based phylogeny of microbial isolates obtained from diseased pea roots.** The phylogenetic tree is based on 67 partial ITS1 sequences from sequenced and type strains. It was constructed using ML method and Tamura–Nei model with 100 bootstrap-value. The isolates used in the specificity assay are highlighted with magenta squares.

**Supplementary Figure 2.** **Clustal omega nucleotide alignment of partial ITS1 sequences from *P. ultimum*, *A. euteiches*, *F. solani*, and *F. oxysporum*.** The consensus sequences for each organism are also included. The colour scale represents sequence similarity across all species included in the alignment: dark green indicates 100% similarity, olive green 80–100%, yellow 60–80%, and grey indicates less than 60%. Boxes show the primer binding regions in the consensus sequences of each organism, in blue F3 primer, in purple B3, in green the F2 region of the FIP primer and in pink the B2 region of the BIP primer. Black dashed boxes indicate the position of the *A. euteiches* qPCR primers used in this study, previously published elsewhere (12).

**Supplementary Figure 3. ITS1 phylogeny** **of partial sequences from *P. ultimum*, *A. euteiches*, *F. solani*, and *F. oxysporum.*** The phylogenetic tree is based on 68 partial ITS1 sequences, the same set used in Supplementary Figure 2. It was constructed using ML method and Kimura model with 100 bootstrap-value. The *F. solani* clade is shown in coral, *F. oxysporum* in lime green, *P. ultimum* in blue and *A. euteiches* in lilac.

**Supplementary Figure 4. *In planta* detection by qPCR. (A-B)** qPCR performed on root gDNA with *A. euteiches* (A) and *F. solani* (B) primers from plants grown in autoclaved vermiculite. **(C-F)** qPCR detection of *A. euteiches* and *F. solani* from roots (C, D) and soil (E, F) gDNA extracted from plants grown in autoclaved soil. **(G-J)** qPCR detection of *A. euteiches* and *F. solani* from roots (G, H) and soil (I, J) gDNA extracted from plants grown in pot soil. All results are based on up to four biological and two technical replicates, with graphs showing mean values at each time point. Note that for all roots samples, 20 ng of gDNA were used as input, whereas 10 ng were used in the soil samples.

**Supplementary Figure 5. *In planta* infections with root rot pathogens (A)** Plants grown in autoclaved vermiculite and **(B)** autoclaved soil evaluated at 2 weeks post inoculation (wpi). **(C)** Plants grown in pot soil evaluated at 4 wpi. Plants were inoculated with *A. euteiches* and *F. solani*, individually and in co-inoculation, with increasing spore concentrations ranging from 10 to 10^5^.

**Supplementary Figure 6. Detection threshold evaluated using PEBBLE across a gradient of pure gDNA concentrations for all target pathogens.** **(A)** Detection of *A. euteiches,* **(B)** *F. solani*, **(C)** *P. ultimum* and **(D)** *F. oxysporum*. The tested gDNA concentrations were 0.02 ng (light blue), 0.2 ng (sky blue), 2 ng (light green), 10 ng (olive green), 20 ng (dark blue) and non-template control (NTC) (black). All reactions were performed at 78 °C for 60 minutes to achieve 66 °C inside the sample, except for *F. solani* where the temperature was increased to 80 °C.

**Supplementary Figure 7. RealAmp assay for detection of *A. euteiches* using magnetic bead-based gDNA extraction.** **(A)** Lab 4 wpi root samples. **(B)** Lab 4 wpi soil samples. All reactions were performed at 66 °C for 60 minutes. Biological replicates were pooled together following gDNA extraction and 5 μl were used per reaction in two technical replicates.

**Supplementary Figure 8.** **RealAmp assay for detection of *A. euteiches* from field samples using column-based gDNA extraction (**DNeasy PowerSoil Pro kit, QIAGEN)**.** All samples listed in Supplementary table 2 were subjected to column base gDNA extraction and evaluated using RealAmp with *A. euteiches* primers. Note that for all roots samples, 20 ng of gDNA were used as input, whereas 10 ng were used in the soil samples.

**Supplementary File 1.** Consensus sequences for *A. euteiches*, *P. ultimum*, *F. solani*, and *F. oxysporum* in FASTA format.

**Supplementary Table 1.** Top ten blastn hits for the LAMP primers targeting *F. solani, F. oxysporum, A. euteiches* and *P. ultimum.*

**Supplementary Table 2.** Summary of the soil baiting experiment results obtained using the plate method described in Materials and Methods.
